# Supplementary material for: Combining Viral Genetics and Statistical Modeling to Improve HIV-1 Time-of-Infection Estimation towards Enhanced Vaccine Efficacy Assessment
Source: Viruses. 2019 Jul 3;11(7):607. doi: 10.3390/v11070607 (PMC6669737; doi:10.3390/v11070607)
Supplement: Supplementary file 1 [file viruses-11-00607-s001.pdf]

Supplementary Material for "Combining Viral Genetics and Statistical Modeling to Improve HIV-1 Time-of-infection Estimation towards Enhanced Vaccine Efficacy Assessment" by Rossenkhan et al.

Supplementary Section A. Theoretical applicability and importance of optimizing an estimator of time since HIV-1 acquisition

To illustrate how the accuracy and precision of an infection time estimator has a major impact on the statistical assessment of correlates of risk within an efficacy trial, we performed power calculations for the AMP efficacy study initiated in 2016 under the two harmonized protocols HVTN 703/HPTN 081 and HVTN 704/HPTN 085 in sub-Saharan Africa and Brazil/Peru/Switzerland/U.S., respectively. Specifically, based on the AMP protocol assumptions about HIV-1 incidence in the placebo group, dropout rate, and pattern of study visits for receiving VRC01 infusions, we studied the power of a statistical procedure for evaluating the null hypothesis that VRC01 concentration has no effect on HIV-1 infection probability, by checking that infections in a VRC01 treated group tend to occur toward the end of the infusion interval, when VRC01 concentration has waned. Specifically, we evaluated a procedure for testing the null hypothesis that among infected cases in the VRC01 treatment group, the mean time between the most recent VRC01 infusion and the HIV-1 acquisition date equals half the average time between infusions among uninfected controls in the same VRC01 treatment group. Rejecting the null hypothesis in the direction of longer mean times in VRC01 group infected cases would support that infections tend to occur when VRC01 concentrations are low, indicating that high VRC01 concentration is associated with lower HIV-1 risk.

These power calculations were done using an HIV-1 infection timing estimator based only on HIV-1 diagnostic testing data, similar to what has been done in previous HVTN efficacy trials, which uses the midpoint of the last negative and first positive diagnosis times. This method is hereafter referred to as the "center of bounds (COB)" estimator. Supplementary Figure 1 shows power curves for rejecting the null hypothesis in favor of larger mean times in VRC01 group infected cases for the analysis done using the COB estimator and the underlying true infection time as the benchmark. The results show 2–3-fold greater statistical power using the true infection times. For example, power increases from 0.43 to 0.97 when the COB-based estimated times vs. true infection times are used when PE in the 30 mg/Kg (10 mg/Kg) VRC01 dose group was estimated to be 78% (54%). Moreover, our work on statistical methods for assessing correlates of HIV-1 infection risk in AMP has shown that it does not appear possible to achieve fully unbiased estimation of correlates or risk parameters based on the COB estimator (data not shown), such that an improved timing estimator is needed for dealing with bias as well as precision when estimating CoR parameters. Incorporating an unbiased timing estimator with low RMSE into the correlates statistical methods will improve their performance.

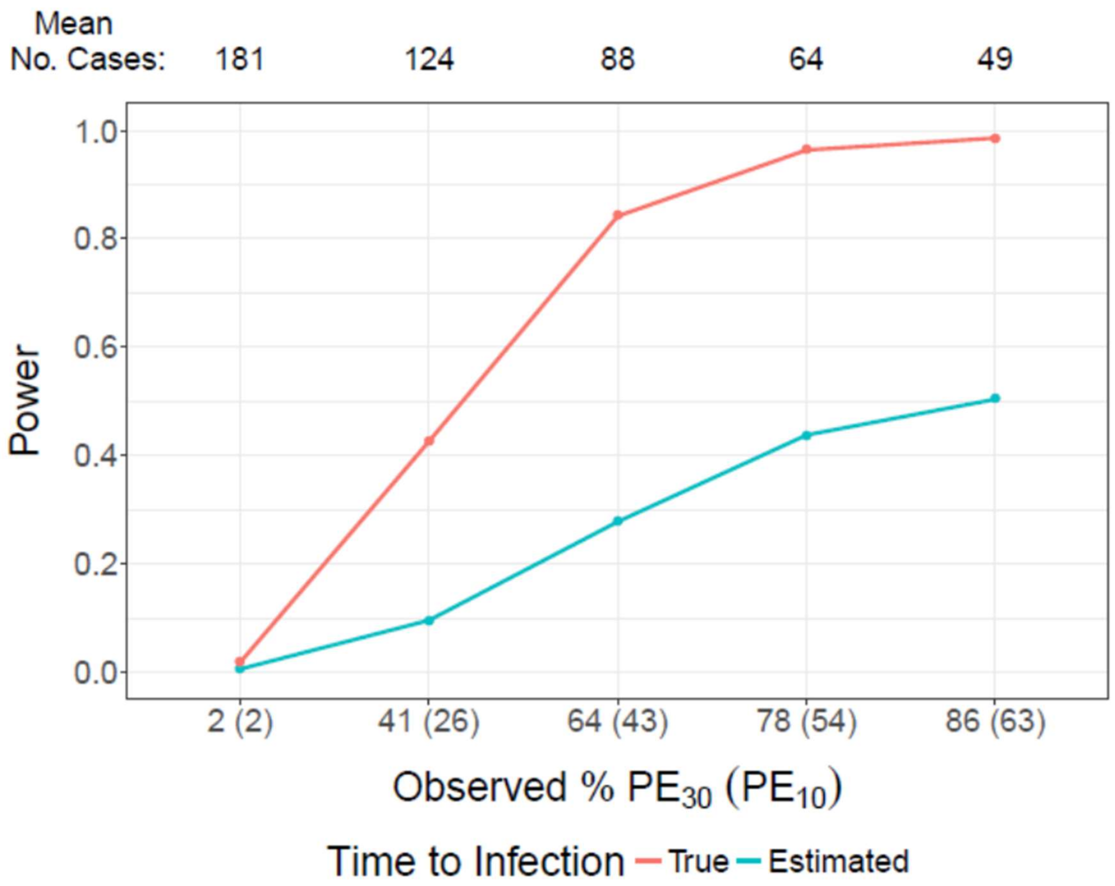

Supplementary Figure 1. Statistical power to detect a time-dependent correlate of risk (CoR) in VRC01 recipients using the true vs. the center of bounds-based estimator of HIV-1 infection time. The x-axis indicates Monte Carlo average prevention efficacy estimates for the 30 mg/Kg (10 mg/Kg) VRC01 dose group vs. placebo corresponding to different effect sizes of the CoR. For each assumed effect size, 500 trials were simulated. The y-axis indicates the proportion of trials with the CoR significantly detected via comparing the mean time between most-recent VRC01 infusion and infection date with one-half the average infusion intervals among controls completing follow-up HIV uninfected using a one-sided Wald test of size 0.025. PE_{30}, prevention efficacy for 30 mg/Kg VRC01 dose group = (one minus ratio (30 mg/Kg VRC01/placebo) of the cumulative probability of HIV-1 infection by 80 weeks post enrollment) × 100%; PE10 is similar for the 10 mg/Kg VRC01 group.

Supplementary Methods Section.

Software pipeline

The software to implement the process shown in Supplementary Figure 2 is implemented in a combination of perl, R, and shell script code, including some scripts that call out to external software hosted online (such as InSites and RAPR) or to local installations of externally supported software (PhyML), modified versions of externally supported software (PFitter), and a local reimplementation of externally supported software (Hypermut 2.0). The pipeline inputs are codon-aligned nucleotide sequences, one set per person and time category, as well as bounds on the time since infection (if available), and optionally plasma viral load measurements (if present these are assumed to be comparable to those used in the calibration training data set, such as when using procedures standardized for HVTN studies). The sequence alignment inputs are processed for evidence of recombination and hypermutation, and altered versions of the inputs are created whenever these features are identified. The outputs of the pipeline, based on these modified inputs, include estimates of infection time from PFitter, PrankenBeast, and COB, estimates of the goodness of fit to a starlike phylogeny model, and various statistics and additional outputs. The phylogenetic trees produced by PhyML are an output that is evaluated manually by eye; here phylogenetic analysis experience is

required to evaluate the trees for quality assurance and reasonable fit, but those trees are not required in any downstream portion of the analysis. Other outputs were used in the calibration of the estimators of multiple-founder infections (Supplementary Tables 1 and 4).

The source code for the infection time and founder multiplicity estimation pipeline is available at https://github.com/pedlefsen/hiv-founder-id, and the pipeline itself has been packaged into a docker container available at https://hub.docker.com/r/philliplab/hiv-founder-id with a working example available in the container that is accessed by running the script /home/docker/example_docker.sh. The container is based on linux with the necessary perl and R packages pre-installed. The command-line interface for the Pre-calibration Step (identify_founders.pl) allows it to be run in one of two modes: in the "standalone" mode, the Pre-calibration Step is run, and then in the Calibration Step, the software returns the results of employing the best calibrated estimator (shifted and scaled versions of PFitter and BEAST outputs) using the parameter fits that we determined in this study (Supplementary Table 2 and 3). When this pipeline is to be applied to data that are similar to one of the data sets that we employed here, we recommend using the best-fitting calibration parameters for the most similar scenario in terms of relevant characteristics such as category of time since infection, HIV-1 subtype, transmission route, and sequencing technology. However, as we note with caution in the Discussion section, further validation is necessary to evaluate robustness of these model fits to other datasets with these or different combinations of the relevant characteristics. In the other mode ("supervised"), the Pre-calibration Step of the pipeline serves as a component of the calibration model parameter estimation pipeline, which can be used for reproduction of our calibration study, or for re-calibration to new or additional data.

Ethics statement

The sequences included in our study were generated following multi-country Institutional Review Board review and was conducted according to the principles expressed in the Declaration of Helsinki. All study participants provided written informed consent for the collection of samples and subsequent analysis.

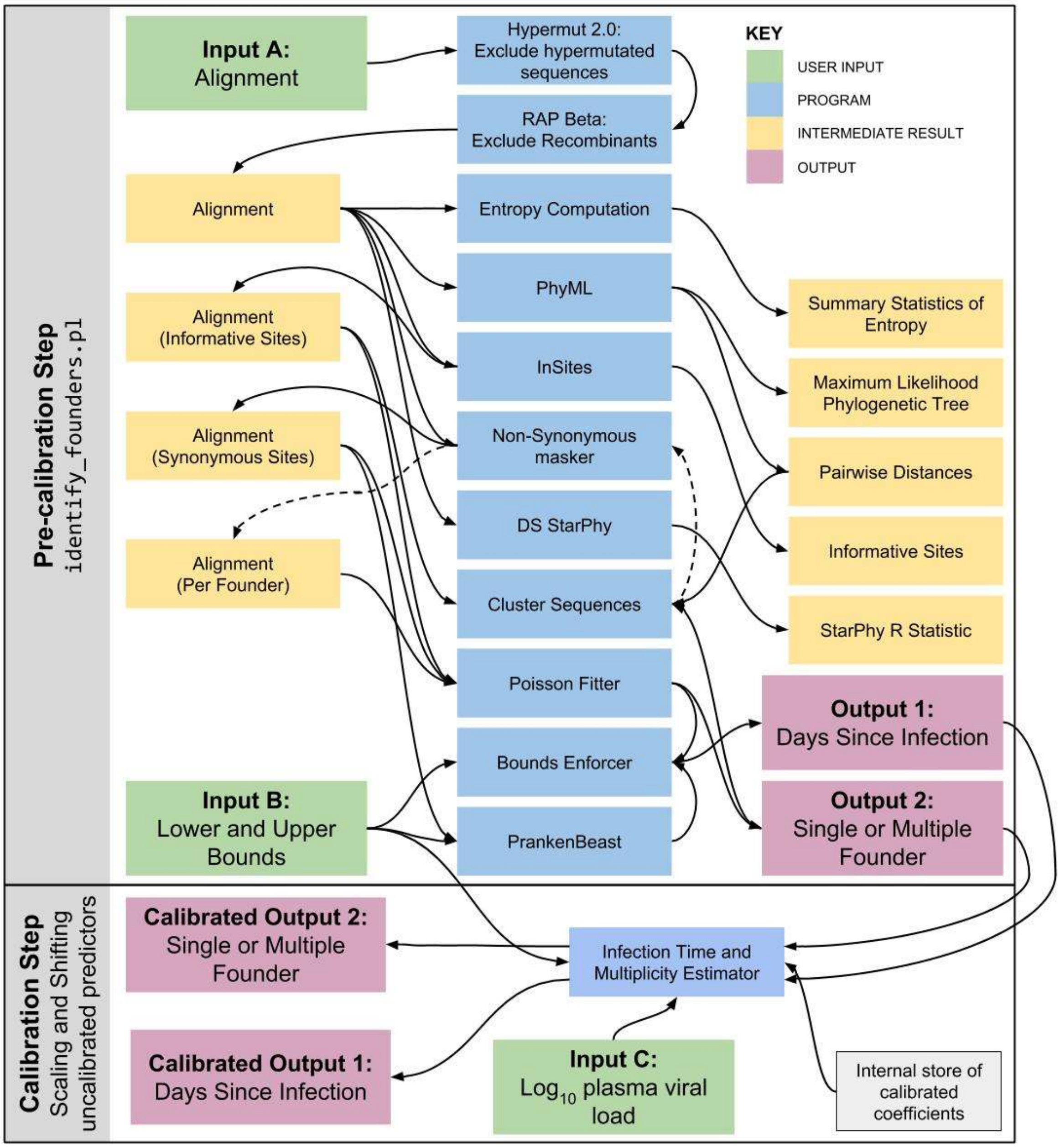

Supplementary Figure 2. hiv-founder-id software pipeline overview. Available from Docker Hub at https://hub.docker.com/r/philliplab/hiv-founder-id. See the description on the repository for instructions. Briefly, the software pipeline accepts sequence data and diagnostic bounds to provide calibrated estimates of both Output 1: Days since infection and Output 2: Multiplicity of infection.

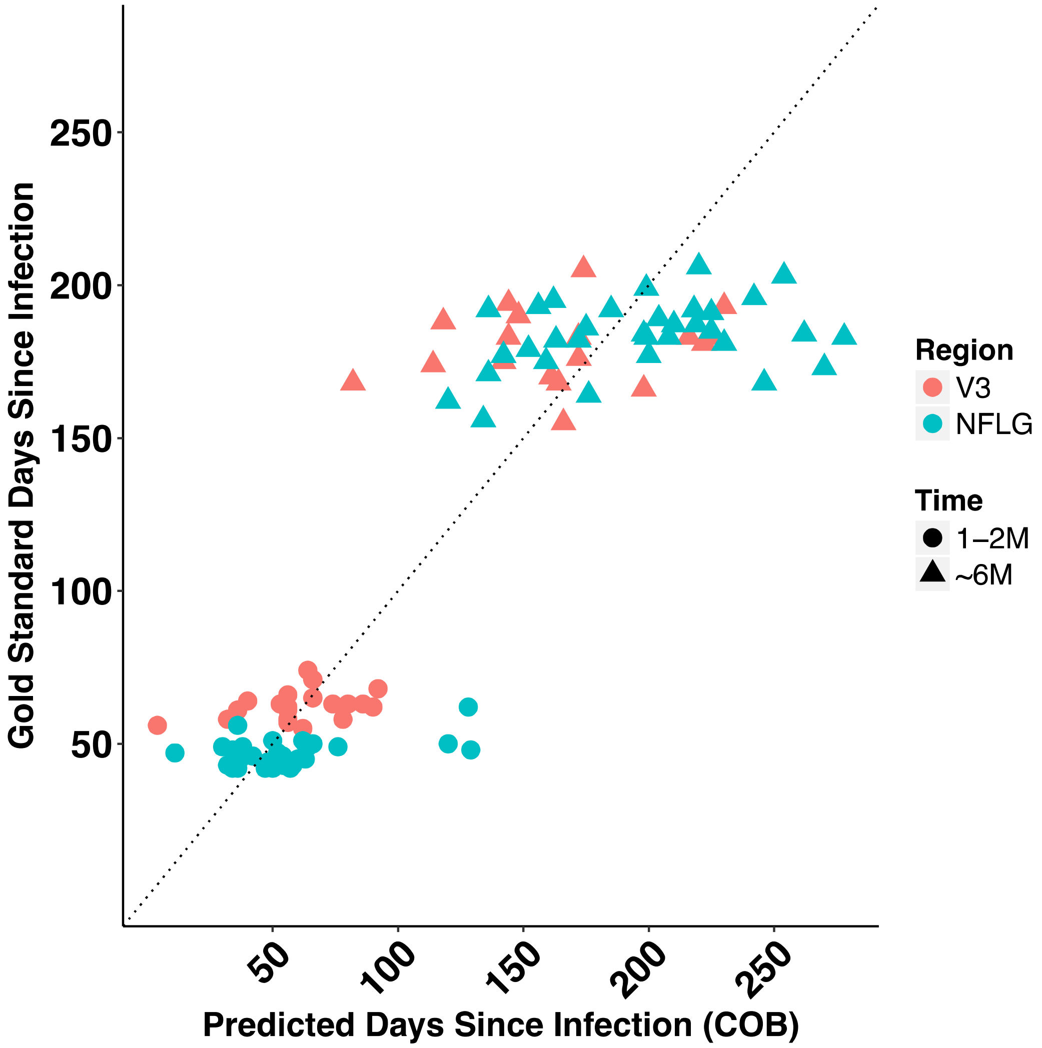

Supplementary Figure 3. Relationship between the center of bounds-predicted value (uncalibrated estimators) and the Gold-standard value for all four of the data sets available for analysis (two studies, each with two time points). Pearson correlations between these values by data set are all positive, but not high: 0.37 (V3 1-2M), 0.18 (V3 ~6M), 0.45 (NFLG 1-2M), 0.35 (NFLG ~6M).

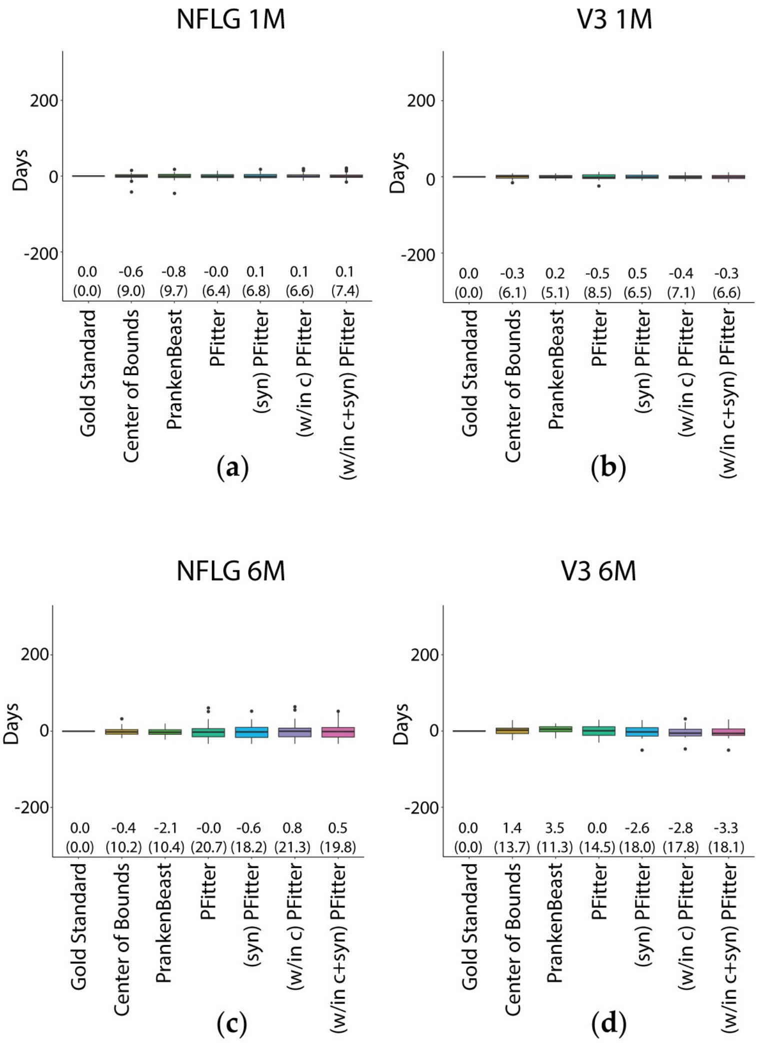

Supplementary Figure 4. Prediction errors of the Center of Bounds, PrankenBeast, Poisson Fitter, and modified Poisson Fitter estimators of infection time after calibration for mutation rate after fitting a Gaussian linear model with terms for $\log_{10}$ plasma viral load (lPVL), the interaction of the estimator with lPVL, the last negative date, the interaction of the estimator with the last negative data, without an intercept. Predictions were made on held out data in a leave-one-out cross-validation scheme (see Methods). The sequences used for prediction were: (a): near full-length genome 1 month; (b): V3 1 month; (c): near full-length genome 6 months; (d): V3 6 months. Box plots are as described for Figure 4.

Supplementary Table 1. Predictors evaluated and selected for calibrated models.

Short name	In Calibrated Time	In Calibrated isMultiple	Long name	Description
lPVL	X	X	Log$_{10}$ plasma viral load	natural logarithm of the plasma viral load at the time of the sample.
bound	**X**	X	upper bound on time since infection	time since the last negative diagnostic test date (accounting for eclipse phase).
time since diagnosis		X	lower bound on infection date	time since diagnosis (accounting for eclipse phase)
diversity		X	mean intra-subject pairwise diversity	average intersequence Hamming distance for all of a subject's sequences at a given time point.
inf.sites.clusters		X	number of clusters	number of clusters when clustering the subalignment of informative sites, as used in w/in clusts PFitter
priv.sites		X	number of private sites	Number of non-informative sites with mutations
DS.Starphy.R		X	DS StarPhy R	Measure of evidence that is not contradictory with a starlike phylogeny
multifounder.Synonymous.				
DS.StarPhy.R		X	(w/in clusts) (syn) DS StarPhy R	Evidence not contradictory with a starlike phylogeny, combining evidence across clusters (non-synonymous codons masked)
inf.sites			number of informative sites	
inf.to.priv.ratio			ratio of informative to private sites	
mean.entropy			average Shannon entropy of ACGT frequencies	
sd.entropy			standard deviation of Shannon entropy of ACGT frequencies	
PFitter.mean.hd			Mean intra-subject pairwise Hamming distance	
PFitter.max.hd			Maximum intra-subject pairwise Hamming distance	
PFitter.chi.sq.stat			Chi squared statistic of PFitter's fits test	
InSites.founders			InSites-based estimate of number of founders	1 if InSites.is.one.founder (ie. if Rolland HVTN estimate is single-founder), otherwise inf.sites.clusters
StarPhy.founders			A hybrid estimator of founder multiplicity	
InSites.is.one.founder			Rolland HVTN estimator of founder multiplicity	1 if diversity and inf.to.priv.ratio exceed thresholds
PFitter.is.poisson			PFitter fits	PFitter output indicating that the Pfitter.chi.sq.stat is significant.
PFitter.is.starlike			PFitter is starlike	PFitter output indicating that the convoluted Hamming distance histogram is nowhere more than 10% different.
Synonymous.PFitter.				
is.poisson			(syn) PFitter fits	PFitter.is.poisson after masking non-synonymous codons.
Synonymous.PFitter.				
is.starlike			(syn) PFitter is starlike	PFitter.is.starlike after masking non-synonymous codons.
multifounder.PFitter.				
is.poisson			(w/in clusts) PFitter fits	PFitter.is.poisson based on modified intersequence distance matrix with inter-cluster sequence distances missing.
multifounder.Synonymous.				
PFitter.is.poisson			(w/in clusts) (syn) PFitter fits	PFitter.is.poisson based on inputs with both (w/in clusts) and (syn) variations.
Synonymous.DS.StarPhy.R			(syn) DS StarPhy R	Evidence not contradictory with a starlike phylogeny (non-synonymous codons masked)
multifounder.DS.Starphy.R			(w/in clusts) DS StarPhy R	Evidence not contradictory with a starlike phylogeny, combining evidence across clusters
v3.not.nflg			data are from CAPRISA 002 study	Indicator that the data source is the Illumuna reeds of the V3 region of clade C infections, versus the other dataset.

Supplementary Table 2. Coefficients of the final selected predictors of Gold-standard infection times in the calibrated models for estimating days since infection. lPVL is the natural logarithm of the plasma viral load. bound is the time since the last negative diagnostic test data. lPVL:est and bound:est are the interactions between the estimate and the lPVL and bound, respectively.

Estimator	Term	Coefficient (95% CI)		
		1M v3	1M nflg	6M (both regions)
**COB**	est	0.00 (-0.77 to 0.77)	-0.29 (-0.58 to -0.00)	0.50 (-0.03 to 1.03)
	Intercept	58.71 (8.03 to 109.39)	60.20 (40.34 to 80.05)	103.28 (1.79 to 204.78)
	lPVL	-0.58 (-5.25 to 4.10)	-1.49 (-3.13 to 0.15)	3.08 (-4.89 to 11.04)
	lPVL:est	0.03 (-0.05 to 0.11)	0.03 (0.00 to 0.05)	-0.02 (-0.06 to 0.02)
	bound	0.02 (-0.57 to 0.62)	0.02 (-0.05 to 0.09)	0.10 (-0.15 to 0.35)
	bound:est	0.00 (-0.01 to 0.00)	0.00 (-0.00 to 0.00)	-0.00 (-0.00 to 0.00)
**PrankenBeast**	est	0.49 (-1.01 to 2.00)	-0.06 (-0.33 to 0.21)	0.88 (0.34 to 1.42)
	Intercept	30.03 (-52.49 to 112.54)	63.10 (44.60 to 81.59)	75.08 (-7.21 to 157.37)
	lPVL	-0.12 (-7.49 to 7.25)	-1.73 (-3.24 to -0.23)	3.70 (-3.44 to 10.85)
	lPVL:est	0.01 (-0.08 to 0.11)	0.02 (0.01 to 0.04)	-0.03 (-0.06 to 0.01)
	bound	0.14 (-0.98 to 1.26)	-0.13 (-0.43 to 0.17)	-0.08 (-0.45 to 0.29)
	bound:est	0.00 (-0.01 to 0.00)	0.00 (-0.00 to 0.00)	-0.00 (-0.00 to 0.00)
**PFitter**	est	0.02 (-0.05 to 0.09)	-0.00 (-0.15 to 0.14)	0.01 (-0.18 to 0.20)
	Intercept	38.60 (12.86 to 64.34)	41.60 (29.55 to 53.66)	188.42 (151.06 to 225.79
	lPVL	2.31 (-0.07 to 4.68)	0.14 (-0.84 to 1.12)	-1.16 (-3.42 to 1.10)
	lPVL:est	-0.00 (-0.01 to 0.00)	0.00 (-0.01 to 0.01)	-0.00 (-0.01 to 0.01)
	bound	-0.02 (-0.18 to 0.14)	0.04 (0.00 to 0.08)	0.02 (-0.10 to 0.13)
	bound:est	0.00 (-0.00 to 0.00)	-0.00 (-0.00 to 0.00)	0.00 (-0.00 to 0.00)
**(syn) PFitter**	est	0.14 (-0.25 to 0.54)	-0.14 (-0.66 to 0.38)	0.24 (-0.50 to 0.98)
	Intercept	36.11 (8.87 to 63.36)	44.61 (31.10 to 58.12)	178.07 (140.66 to 215.49)
	lPVL	2.97 (0.30 to 5.64)	0.01 (-1.05 to 1.07)	-0.80 (-2.89 to 1.28)
	lPVL:est	-0.02 (-0.05 to 0.01)	0.00 (-0.04 to 0.04)	-0.01 (-0.05 to 0.03)
	bound	-0.07 (-0.26 to 0.11)	0.02 (-0.03 to 0.07)	0.06 (-0.05 to 0.17)
	bound:est	0.00 (0.00 to 0.00)	0.00 (-0.00 to 0.00)	-0.00 (-0.00 to 0.00)
**(w/in clusts) PFitter**	est	0.03 (-0.32 to 0.37)	-0.03 (-0.39 to 0.33)	-0.03 (-0.29 to 0.22)
	Intercept	46.87 (17.76 to 75.97)	42.51 (26.49 to 58.52)	192.38 (157.32 to 227.44
	lPVL	1.07 (-1.68 to 3.82)	0.03 (-1.25 to 1.31)	-1.33 (-3.39 to 0.73)
	lPVL:est	0.00 (-0.03 to 0.03)	0.00 (-0.02 to 0.03)	0.00 (-0.01 to 0.01)
	bound	0.03 (-0.19 to 0.25)	0.04 (-0.01 to 0.10)	0.02 (-0.08 to 0.12)
	bound:est	0.00 (0.00 to 0.00)	-0.00 (-0.00 to 0.00)	0.00 (-0.00 to 0.00)
**(w/in clusts) (syn) PFitter**	est	0.95 (-0.39 to 2.30)	-0.46 (-1.45 to 0.54)	0.30 (-0.53 to 1.14)
	Intercept	31.20 (-1.04 to 63.45)	48.86 (32.35 to 65.37)	178.16 (144.27 to 212.05)
	lPVL	2.07 (-0.54 to 4.68)	-0.27 (-1.58 to 1.04)	-0.84 (-2.80 to 1.13)
	lPVL:est	-0.06 (-0.16 to 0.04)	0.02 (-0.06 to 0.10)	-0.01 (-0.06 to 0.04)
	bound	0.08 (-0.13 to 0.29)	0.00 (-0.05 to 0.06)	0.06 (-0.05 to 0.16)
	bound:est	0.00 (-0.01 to 0.01)	0.00 (-0.00 to 0.01)	-0.00 (-0.00 to 0.00)

Supplementary Table 3. Coefficients of the calibrated models that estimate days since infection using the estimator alone, without an intercept term, lPVl, or bounds. Note that although the diagnostic bounds are not included in the calibration model, the results are bounded by the diagnostic bounds dates.

Estimator	Term	Coefficient (95% CI)		
		1M v3	1M nflg	6M (both regions)
**COB**	est	0.94 (0.79 to 1.08)	0.73 (0.62 to 0.83)	0.93 (0.88 to 0.99)
**PrankenBeast**	est	0.74 (0.66 to 0.81)	0.49 (0.40 to 0.57)	0.81 (0.76 to 0.86)
**PFitter**	est	0.08 (0.02 to 0.15)	0.23 (0.13 to 0.33)	0.54 (0.43 to 0.65)
**(syn) PFitter**	est	0.48 (0.14 to 0.82)	1.04 (0.66 to 1.42)	2.03 (1.48 to 2.57)
**(w/in clusts) PFitter**	est	0.45 (0.21 to 0.69)	0.40 (0.25 to 0.56)	0.82 (0.63 to 1.01)
**(w/in clusts) (syn) PFitter**	est	0.94 (0.30 to 1.59)	1.70 (1.18 to 2.23)	2.65 (1.90 to 3.41)

Supplementary Table 4. Coefficients in the calibrated model for estimating founder multiplicity.

Short name	Long name	Coefficient (95% CI)	Description	Variance Explained
**intercept**	intercept	-3.50 (-9.35 to 1.90)	an offset (on the logistic scale) to the probability of isMultiple	0.00%
**lPVL**	Log_{10} plasma viral load	0.83 (0.27 to 1.54)	natural logarithm of the plasma viral load at the time of the sample.	0.00%
**bound**	upper bound on time since infection	0.00 (-0.01 to 0.02)	time since the last negative diagnostic test date.	0.00%
**time since diagnosis**	lower bound on infection date	0.02 (-0.00 to 0.03)	time since diagnosis. (accounting for eclipse phase)	0.51%
**diversity**	mean pairwise diversity	-1206.97 (-1929.46 to -739.47)	average intersequence Hamming distance.	37.97%
**inf.sites. clusters**	number of clusters	0.70 (0.08 to 1.59)	number of clusters when clustering the subalignment of informative sites, as used in w/in clusts PFitter	0.60%
**priv.sites**	number of private sites	-0.03 (-0.06 to -0.01)	Number of non-informative sites with mutations	4.20%
**DS.Starphy.R**	DS StarPhy R	2.18 (-1.76 to 6.32)	Measure of evidence that is not contradictory with a starlike phylogeny	18.84%
**multifounder. Synonymous. DS.StarPhy.R**	(w/in clusts) (syn) DS StarPhy R	-6.23 (-12.68 to -1.31)	Evidence not contradictory with a starlike phylogeny, combining evidence across clusters (non-synonymous codons masked)	12.48%
